# The Biological Influence and Clinical Relevance of Polymorphism Within the NKG2D Ligands

**DOI:** 10.3389/fimmu.2018.01820

**Published:** 2018-08-16

**Authors:** Jianmin Zuo, Fiyaz Mohammed, Paul Moss

**Affiliations:** Institute of Immunology and Immunotherapy, University of Birmingham, Birmingham, United Kingdom

**Keywords:** polymorphism, single nucleotide, NKG2D ligands, binding affinity, cytotoxicity, immunologic, natural killer cells

## Abstract

NKG2D is a major regulator of the activity of cytotoxic cells and interacts with eight different ligands (NKG2DL) from two families of MIC and ULBP proteins. The selective forces that drove evolution of NKG2DL are uncertain, but are likely to have been dominated by infectious disease and cancer. Of interest, NKG2DL are some of the most polymorphic genes outside the MHC locus and the study of these is uncovering a range of novel observations regarding the structure and function of NKG2DL. Polymorphism is present within all NKG2DL members and varies markedly within different populations. Allelic variation influences functional responses through three major mechanisms. First, it may drive differential levels of protein expression, modulate subcellular trafficking, or regulate release of soluble isoforms. In addition, it may alter the affinity of interaction with NKG2D or modulate cytotoxic activity from the target cell. In particular, ligands with high affinity for NKG2D are associated with down regulation of this protein on the effector cell, effectively limiting cytotoxic activity in a negative-feedback circuit. Given these observations, it is not surprising that NKG2DL alleles are associated with relative risk for development of several clinical disorders and the critical role of the NKG2D:NKG2DL interaction is demonstrated in many murine models. Increased understanding of the biophysical and functional consequences of this polymorphism is likely to provide insights into novel immunotherapeutic approaches.

## Introduction

NKG2D is a dominant activating receptor on cytotoxic lymphocytes, including natural killer (NK) cells, γδ T cells, NKT cells, and αβ T cell subsets (1). Engagement of NKG2D with NKG2D ligands (NKG2DLs) on target cells triggers cytotoxicity or cytokine production and plays an important role in both innate and adaptive immune responses, including control of viral infection, tumorigenesis and pathogenesis of autoimmune diseases (2–4).

A striking feature of the NKG2D:NKG2DL interaction is that only a single gene encodes NKG2D while there are eight *NKG2DL* genes within the human genome. These NKG2D ligands comprise six cytomegalovirus glycoprotein UL16 binding proteins and two major histocompatibility complex class I polypeptide-related sequences (MICA/B). In mice, NKG2D ligands comprise five RAET1 family members, three H60 proteins and MULT-1 (5–8). MICA and MICB contain α1, α2, and α3 domains together with a transmembrane domain and short cytoplasmic tail. In contrast, ULBP ectodomains comprise only the MHC-like α1 and α2 domain and ULBP1, ULBP2, ULBP3, and ULBP6 are GPI-anchored receptors while ULBP4 and ULBP5 encompass a membrane anchor and cytoplasmic tail (9).

**Figure 1:**
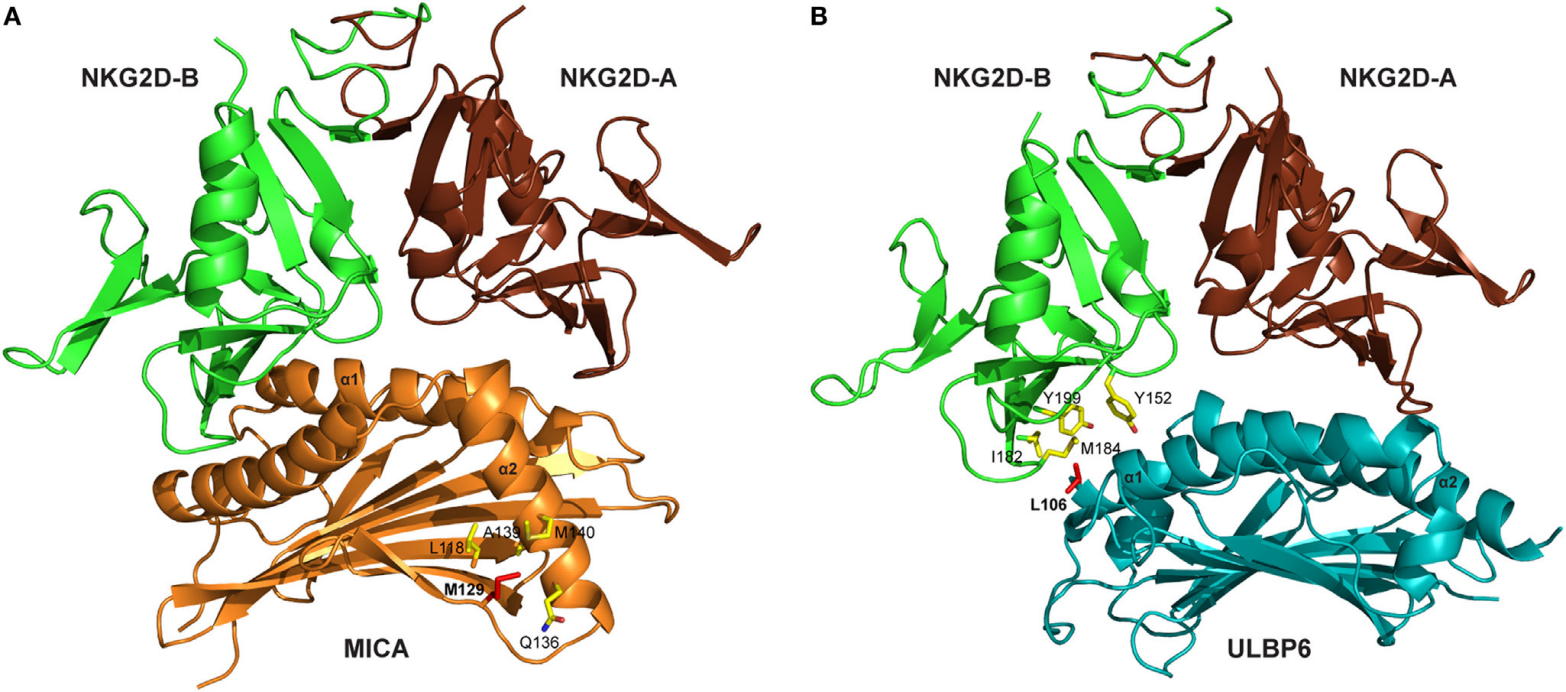
Crystal structures of NKG2D-ligand complexes. **(A)** Ribbon representation of the MICA–NKG2D complex [PDB code 1HYR, Li et al. (10)]. NKG2D homodimer [NKG2D-A (brown) and NKG2D-B (green)] interacts with the α1 and α2 domains of monomeric MICA (orange). The clinically relevant polymorphic residue Met129 (red) in MICA is located distal to the MICA–NKG2D interface. Partially buried Met129 mediates non-polar interactions with MICA residues (ball and stick format) that protrude from the α2 helix. The α3 domain of MICA (residues Thr181-Ser274) has been omitted. **(B)** Ribbon representation of the ULBP6–NKG2D complex [PDB code 4S0U; Zuo et al. (13)]. NKG2D homodimer [NKG2D-A (brown) and NKG2D-B (green)] interacts with the α1 and α2 domains of ULBP6 (teal). The disease-associated polymorphic residue Leu106 (red) in ULBP0602 is in close proximity to the ULBP6–NKG2D docking interface, inserting directly into the NKG2D hydrophobic pocket lined by several non-polar residues (ball and stick format). The figure was generated with PyMOL (Molecular Graphics System, Version 2.0 Schrödinger, LLC).

Several crystal structures of the NKG2D/NKG2DL interaction have been resolved, including NKG2D–MICA (10, 11), NKG2D–ULBP3 (12), and NKG2D–ULBP6 (13). These reveal that the symmetric NKG2D homodimer binds to a monomeric NKG2D ligand *via* a diagonal mode of engagement which is similar to TCR–pMHC interaction. In particular, the saddle-shaped NKG2D homodimer sits astride the NKG2D ligand helices with the NKG2D monomers A and B focused on the NKG2D ligand α2 and α1 helices, respectively (Figure 1) (13).

DAP10 is the binding partner for NKG2D and ligation and cross-linking of NKG2D leads to the recruitment of the p85 subunit of PI3K and Grb2-Vav1-Sos1 complex to the phosphorylated DAP10. This elicits the phosphorylation of the kinases Jak2, Stat5, Akt, MEK1/2, and Erk. This in turn induces calcium flux, actin reorganization, degranulation and, finally, cytotoxicity to target cells. PI3K inhibitors can thus completely block cytotoxic activity (14).

All *NKG2DL* genes demonstrate considerable polymorphism (15–18) and this variation is present within each of the domains and with a relatively random distribution, a feature in contrast to the focused variation that is observed with MHC class I genes (19, 20). Polymorphism is most significant within the *MICA* and *MICB* genes (17) where 62 single nucleotide polymorphisms (SNP) and 25 SNPs have been identified, respectively. In relation to the ULBP gene family, 28, 12, 10, 20, 10, and 14 SNPs have been reported within ULBP1-6 (15, 17, 21, 22). Interestingly, polymorphisms within MICA and MICB are distributed throughout the three extracellular α1–α3 domains and there are no identified coding polymorphisms within residues 40–89 within the section of the α1 domain that is predicted to mediate binding with NKG2D (9, 23). This profile is somewhat different to the profile of ULBP polymorphism where several variants are predicted to influence directly the site of NKG2D binding.

The frequency of individual *MICA* and *MICB* alleles varies in different populations around the world. In particular, MICA*008 is the most frequent allele worldwide (9) (except in South American Indians) (24) followed by MICA*002, MICA*010, MICA*009, and MICA*004. The *MICB* gene is more homogeneous, with MICB*005 being the most common allele and less representation from MICB*002, MICB*004, MICB*008, and MICB*014 (9). Population-based variations in polymorphism are also a dominant feature and with wide variation in the frequency of SNPs within *ULBP* in Euro-Caucasoid, Afro-Caribbean, and Indo-Asian individuals (15) and most variation focused within *ULBP3, ULBP4*, and *ULBP6* (15, 16).

## Polymorphism within NKG2D Ligands Reflects the Impact of Differential Selective Forces within Evolution

An important question within NKG2DL biology relates to the evolutionary pressures that have driven gene duplication and polymorphism within MIC and ULBP family members. The expression of each gene is differentially regulated within tissues and it is likely that temporal and tissue-specific regulation of NKG2DL has served to optimize control of infectious challenges while limiting the development of autoimmune complications (16). Evidence for this effect may be seen in relation to the ULBP0601 and ULBP0602 proteins, where ULBP0601 homozygosity is associated with increased cytotoxic activity in comparison to the ULBP0602 variant (13). Viral infections may have represented a particularly important selective force as viral immunoevasins generally bind only to specific ligands and no single viral immunoevasin has yet been described which can bind to all NKG2DL family members.

Assessment of NKG2DL polymorphism within different species might also provide some clues as to the pace and potential determinants of gene diversification. Meyer et al. (25) sequenced a range of *MIC* genes from non-human primates and demonstrated that these most likely derive from a single common MICB-like ancestor (26). Much of the polymorphism within ULBP genes appears to have arisen very early in the development of *Homo sapiens* and prior to migration out of Africa (15, 27). However, the pattern of diversity varies markedly across the globe and is likely to reflect differential selection to pathogens within different environments (9, 24). Some inferences may be drawn from the evolution of the KIR genes where, in addition to infection, a role in mediating successful pregnancy outcome also appears to be important (28).

## The Clinical Importance of Polymorphism within NKG2D Ligands

Polymorphisms within *NKG2DL* alleles have been identified as risk factors for a range of different clinical disorders. As discussed above, NKG2DL expression is increased by inflammatory stimuli (29) and as such the strong association of *ULBP6* SNP (rs9479482) with Alopecia Areata is compatible with its autoimmune pathogenesis (AA) (30, 31). Indeed, NKG2D^+^ T cells have been identified as important mediators in the initiation of this disease (32). The first association of MICA polymorphism with autoimmune disease was observed by Mizuki et al. (33) in Behçet’s disease and this has been followed by 12 further reports (Table 1).

**Table 1:** The clinical relevance of polymorphism within NKG2D ligands.

	NKG2DL	Single nucleotide polymorphisms	Diseases	Reference
Malignancy association	MICA	129-Met/Val	Nasopharyngeal carcinoma	Douik et al. (34)
	MICA	MICA-5.1	Oral squamous cell carcinoma	Tamaki et al. (35, 36)
	MICA	MICA-5.1	Breast cancer	Lavado-Valenzuela et al. (37)
	MICA	213 thr and 251 gln	Cervical cancer	Jumnainsong et al. (38)
	MICA	rs2596542, rs2596538	Hepatitis C virus (HCV)-induced hepatocellular carcinoma (HCC)	Kumar et al. (39); Lo et al. (40); Goto et al. (41)
	MCIA	rs2596542G/A 129Met/Val; 251Gln/Arg 175Gly/Ser; triplet repeat	Hepatitis B virus-induced hepatocellular carcinoma	Tong et al. (42)
Virus infection	MICA	MICA-5.1	CMV reactivation in HIV-infected patients	Moenkemeyer et al. (43)
	MICB	rs3132468	Dengue shock syndrome and non-severe dengue	Khor et al. (44); Whitehorn et al. (45); Dang et al. (46)
Autoimmune diseases	MICA	129-Met/Val	Chronic Chagas heart disease	Ayo et al. (47)
	MICA	129-Met/Val	Ankylosing spondylitis	Amroun et al. (48)
	MICA	rs1051794	Rheumatoid arthritis	Kirsten et al. (49)
	MICA	129-Met/Val	Inflammatory bowel disease	Lopez-Hernandez et al. (50); Zhao et al. (51)
	MICA	129-Met/Val	Lupus erythematosus	Yoshida et al. (52)
	MICA	129-Met/Val	Type I diabetes	Raache et al. (53)
	MICA	129-Met/Val	Psoriatic disease	Pollock et al. (54)
	MICA	Triplet repeat microsatellite	Behçet disease	Mizuki et al. (33)
	MICA	MICA-A9 triplet repeat	Psoriatic arthritis	Gonzalez et al. (55)
	ULBP6	Rs1543547	Diabetic nephropathy	Mcknight et al. (56)
	ULBP6	Rs9479482	Autoimmune alopecia	Petukhova et al. (30)
HSCT	MICA	129-Met/Val	Chronic GvHD	Boukouaci et al. (57)
	ULBP6	ULBP0601/ULBP0602	HSCT overall outcome	Antoun et al. (58)
	MICA	129-Met/Val	HSCT clinical outcome	Isernhagen et al. (59)

Viral infection is a potent stimulus for NKG2DL expression and an early report identified that MICA polymorphism was associated with increased risk of CMV reactivation in HIV-seropositive patients (43). Strikingly, genome-wide association study analysis of 2,008 patients with pediatric Dengue shock syndrome and 2,018 controls from Vietnam demonstrated that the *MICB* polymorphism rs3134899 was one of only two associated risk alleles with a per-allele odds ratio of 1.34 (44). Moreover, a replication study in Thai patients confirmed these findings (46), which were also apparent in patients with less severe clinical phenotypes of dengue as well as an infant group (45, 46). As such, the association of *MICB* alleles with dengue is one of the most well characterized allelic associations of a non-HLA gene with any infectious disease.

Cancer may be been an important selective force in NKG2DL evolution and expression is increased markedly during cell transformation. Several reports have demonstrated an association between NKG2DL alleles and a range of different cancer subtype and it will be of interest to see if this is replicated in future studies (34–42). Tumor-specific immune responses are also central in disease control and are exemplified in stem cell transplantation (HSCT) (60) where the curative effect relies mainly on the graft versus leukemia effect. NKs contribute to alloreactive responses (61, 62) and NKG2DL polymorphism appears to play a particularly important role in this setting (63). Again, the MICA-129 Met/Val dimorphism is informative with higher relapse rates observed in association with the MICA-129Met/Met homozygous genotype (57) and increased incidence of cGVHD with MICA-129 Val/Val homozygotes (59). This has been interpreted as reflecting stronger NK and CD8^+^ T cell activation in the presence of the MICA-129Met allele although the longer term effects may be mitigated by subsequent down regulation of NKG2D on effector cells. We also investigated the impact of ULBP in 371 SCT patient–donor pairs and related this to clinical outcome (58), observing a strong association between the ULBP0602 allele and overall survival. This effect might reflect either the direct consequence of cytotoxic activity of NK and T cells against tumor cells or a regulatory role of the NK subset on subsequent development of the alloreactive T cell response (64–67).

## The Functional Impact of Polymorphism within NKG2D Ligand Proteins

A major ambition is now to understand the biological importance of physiological variation within the NKG2DL proteins and how this information may be used both to understand established disease associations and to potentially develop novel immunotherapeutic approaches.

**Figure 2:**
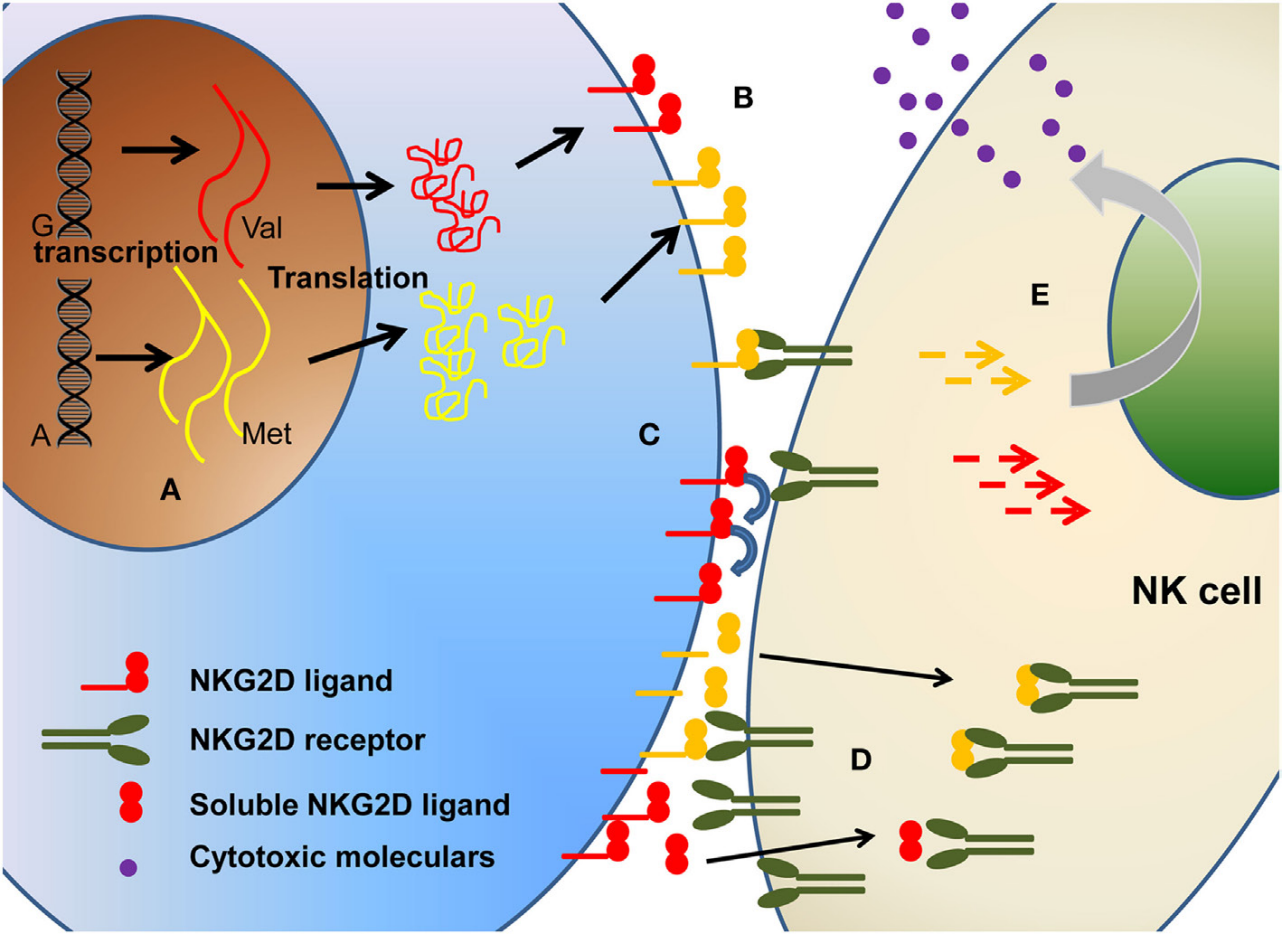
The polymorphism of NKG2D ligands will affect the biological function of NKG2D ligands in multiple levels, including **(A)** different transcription, **(B)** different expression level on cell surface, **(C)** different binding affinity, **(D)** different soluble ligands shedding. Collectively, these will ultimately affect the **(E)** downstream signaling and cytotoxicity function.

In this review, we address this challenge in relation to the influence of polymorphism (Figure 2) on (1) expression level of NKG2DL proteins, (2) differential affinity for NKG2D, and (3) modulation of cytotoxic activity.

### The Influence of Polymorphism on the Expression of Surface and Soluble Forms of NKG2DL

Perhaps the most immediate influence of NKG2DL polymorphism is that it may modulate the magnitude of protein expression at the cell surface. Indeed, use of a functional genomics system whereby a single copy of MICA cDNA can be stably integrated into CHO cells revealed differential transcriptional activity that varied by sixfold across four different alleles and correlated with protein expression (68). Two MICA polymorphisms associated with increased risk of HCV-related hepatocellular cancer are both located at the 5′ flanking region of MICA, rs2596542 (39) being 4.7 kb and rs2596538 (40) 2.8 kb upstream of the *MICA* gene. Of interest, the rs2596538 allele is located at a binding site for transcription factor specificity protein 1 and both SNPs modulate the level of soluble MICA protein due to relative transcriptional activity at the MICA locus (39, 40). The rs1051792 SNP of the MICA gene resulting in the MICA-129Met/Val dimorphism was the first MICA polymorphism for which a functional consequence was described and the MICA-129Met variant is associated with particularly strong NKG2D engagement. The effect of this exchange is more subtle, in that the MICA-129Met variant is associated with increased transcriptional activity but protein retention within intracellular compartments (69) and higher serum MICA levels are seen in patients with hepatitis B who are homozygous for the MICA-129Val allele (42). As such, polymorphism can influence not only the magnitude of protein expression but also the pattern of expression within the cell. Our own studies have focused on polymorphism within ULBP6 (13) where stably integrated CHO cell lines with a single copy of the ULBP0601 and ULBP0602 alleles, and primary patient samples, did not reveal any difference in surface expression or shedding according to genotype.

As such, polymorphisms within NKG2DLs that act to modulate protein expression, subcellular location, and surface shedding are important regulators of differential NKG2DL activity.

### The Importance of NKG2DL Polymorphism on Binding Affinity to NKG2D

Engagement of NKG2D ligands with NKG2D is a crucial step in the activity of many cytotoxic cells. Again, study of the MIC-A129 Met/Val dimorphism has been crucial in studies of how this may be influenced by allelic variation. Steinle et al. transfected a range of nine different MICA alleles into a reporter cell line and, after determining equivalent levels of cell surface expression, then interrogated their binding to soluble NKG2D. A hierarchy of fluorescent intensity was observed, with 5 alleles demonstrating stronger binding compared to the other 4 (70). Interestingly, the presence of methionine at position 129 (Met129) was the sole determinant of strong binding. In subsequent work, Isernhagen and colleagues expressed two MICA*0701 variants, with either methionine (the wild-type amino acid) or valine at position 129, and examined NKG2D affinity on a range of different cell types. The slope of NKG2D engagement in relation to increasing MICA expression intensity was steeper for the MICA-129Met variant, indicating higher avidity compared to the MICA-129Val variant (59). Based on the crystal structure of MICA*01 (10), this biallelic position does not participate directly in the MICA–NKG2D interface but may indirectly modulate NKG2D binding *via* a conformational change (Figure 1A). The methionine side chain protrudes from the β-sheet base and mediates an extensive network of non-polar interactions with Gln136, Ala139, and Met140 from the N-terminal helical stretch of α2, a region that is in close proximity to the MICA–NKG2D interface (11). It is conceivable that this conformation within the MICA-Met129 variant permits optimal contacts with bound NKG2D which is presumably lost in the MICA-Val129 form and this may account for the differing ligand binding affinities associated with the MICA-129Met/Val dimorphism.

We have also examined the binding of NKG2D to recombinant forms of the two major ULBP6 alleles (ULBP0601 and ULBP0602), using surface plasmon resonance (13). Strikingly, ULBP0602 demonstrated a very high affinity for its ligand with fast binding and slow dissociation. Indeed, the measured affinity of 15.5 nM is over 10-fold higher than the equivalent binding for ULBP0601 and substantially greater than any other ULBP family member. The structure of the NKG2D/ULBP0602 interaction revealed hydrophobic contacts at the ULBP0602/NKG2D interface sufficient to explain this difference as position Leu106 in ULBP0602, located at the NKG2D receptor ligand interface, inserts directly into the center of the hydrophobic patch B of NKG2D, forming numerous non-polar contacts with surrounding residues (Tyr152, Ile182, Met184, and Tyr199) (Figure 1B). In contrast, ULBP0601 has a charged and lengthy Arg at this position and its introduction within this predominantly hydrophobic environment is likely to be detrimental for NKG2D binding.

Collectively, these studies reveal that variation in the affinity of the interaction with NKG2D is likely to have acted as an important selective force in the polymorphism within NKG2D ligands.

### Polymorphism Within NKG2DL Have an Important Influence on Cytotoxic Activity of Effector Cells

The most important functional outcome of NKG2DL:NKG2D engagement is the degree of cytotoxic activity from the effector cell. A range of studies (13, 59, 68) have indicated subtle differences in NKG2DL structure can translate into significant variation in the cytotoxic capacity of effector cells. This has demonstrated clearly within the MIC-A family by increased levels of NK cell cytotoxicity and cytokine production following engagement with cells expressing the high-affinity MICA-129Met allele in comparison to those with surface expression of MICA-129Val (59). A comparable profile was observed in relation to co-stimulation of CD8^+^ T cells where, again, the 129Met allele provided stronger signaling to NKG2D^+^ CD8^+^ cells in concert with TCR engagement.

In light of these findings it might be considered that the evolution of high-affinity NKG2DL alleles would result inexorably into positive selection within a population. However, as almost always in biology, there is clearly a balance in the relation to the optimal affinity for NKG2DL:NKG2D binding. In particular, as the level of NKG2DL protein expression increases on the surface of a target cell, as might occur in the setting of viral infection or transformation, this can lead to dramatic down regulation of NKG2D expression on the effector cell in situations where the NKG2DL ligand has a high affinity for NKG2D. As such, alleles such as MICA-129Met might offer a cytotoxic advantage against targets with low levels of NKG2D expression but become counterproductive as they drive the “NKG2D exhaustion” of effector cells following high levels of expression due to cellular stress.

Striking differences are also observed in the cytotoxic capacity of NK cells taken from different donors (68). A comparison of effector cell activity from 22 healthy donors revealed marked interindividual variation in the cytotoxic ability and, surprisingly, this was correlated both positively and negatively to MICA expression level in different donors. The authors argue that individual responses are “tuned” to different “dose bandwidths” of NKG2DL expression, again challenged the simple hypothesis that higher receptor levels promote greater responsiveness.

We also observed that effector cells expressing the high-affinity ULBP6 allele, ULBP0602, elicited weaker killing from a range of effector NK and T cells compared to ULBP0601, and was correlated with less downregulation of NKG2D (13). We suggest that this may result either from the ultra stable binding nature of the NKG2D/ULBP0602 interaction, which equates to a *t*_1/2_ of ~550 s at 25°C, acting to limit serial triggering of NKG2D receptors (71, 72). Alternatively, shedding of the high-affinity soluble ULBP0602 can inhibit NKG2D binding to the NKG2D ligands on the cell surface.

### Exploiting the Translational Potential of Polymorphism Within NKG2DL

The reports of polymorphism within NKG2D ligands, particularly MICA and ULBP6, and disease risk indicate that NKG2DL family members may represent an important therapeutic targets for treatment. The interaction of NKG2D and NKG2D ligands has been shown to be particularly important in the control of malignant disease. *In vivo* studies in murine models have shown that expression of NKG2D ligands on tumor cells is an important determinant of tumor control (73) and as such it is no surprise that tumor progression is associated with selection for tumor variants which are able to evade NKG2D-mediated immune recognition. In the setting of inflammatory disease, one potential strategy might be to downregulate NKG2D receptor expression on T cells and NK cells through soluble NKG2D ligands or immune suppressive cytokine (74, 75). In the setting of malignant disease, considerably more information is required on the profile of NKG2DL expression within individual tumor and how this is related both to the profile of somatic mutations within the tumor and the tumor microenvironment. Cytokines such as TGF-β have also been shown to regulate NKG2DL expression and represent a further influence on ligand regulation within the tumor microenvironment (76).

As such, interventions that can modulate the functional outcome of the NKG2D:NKG2D interaction may represent important and novel immunotherapeutic approaches (77). This indicates the requirement for continuing research to understand and exploit the lessons that can be derived from the extensive polymorphic variation within this remarkable ligand interaction.

## Author Contributions

All authors listed have made a substantial, direct, and intellectual contribution to the work and approved it for publication.

## Conflict of Interest Statement

The authors declare that the research was conducted in the absence of any commercial or financial relationships that could be construed as a potential conflict of interest.
